# 

**Published:** 1878-05-20

**Authors:** 


☞All communications relating to the business of The Record, for the years 1877 and 1878, must be addressed to DR. R. C. WORD, Managing Editor Southern Medical Record, Atlanta, Ga.

☞Brief and practical communications are solicited on all subjects pertaining to medicine; also reports on cases in practice.

☞Send money by check, postal order or registered letter.

☞Write your name, post-office, county and State plainly.

### TO OUR SUBSCRIBERS.

Those in arrears for subscription will please remit at once, and oblige,

R. C. Word,
Managing Editor.

Receipted.—Drs. J A Ashford, Z T Young, E K Bozeman, J L Hamilton, W C Moore, M V B Miller, J T Mooty, J W Day, R A Sells, L C Harvey, A M Winn, W J Sterling, J W Fair, 6ms., W H Roberts, A A Stanley, A D Sanders, J B Hughes, A G Groves, A C Simonton, J D Moon, R Inge, L B Bouchelle, E C Anderson, J A McCallum, C D Tatman, C C Jones, J W Strong, J C Moody, J D Harrell, R W Lovett, L G Hardeman, J N Wardsworth, S L Lockwood, B M Walker, R Collins, A W Agnew, G L Mills, Library Surgeon Gen'l, B F Rudisill, W P Anderson, William Sellers, B J Foster, B E Clark, Jno J Gage, J R Phillips, T B Swift, O M Doyle, J T Stoddard, G L Sanders 6ms., C P Sanders, W G Hays, L W Coleman, E H Wright, T F Green, A J Ellis, M Giesy, E A Speed, C W McDaniel, J A Allen, P S Anderson. C G Nichols, W H Wilson, Nisbit & Nisbit.

